# Blood Glucose Levels and Stroke Severity in Patients with Acute Small Subcortical Infarct

**DOI:** 10.3390/brainsci15121278

**Published:** 2025-11-28

**Authors:** Stefano Mombelli, Chiara Rinaldi, Andrea Morotti, Stefano Forlivesi, Chiara Bassi, Marialuisa Zedde, Rosario Pascarella, Giuseppe Reale, Pietro Caliandro, Aurelia Zauli, Federico Mazzacane, Beatrice Del Bello, Andrea Zini, Francesco Arba

**Affiliations:** 1 Neurofarba Department, University of Florence, Careggi University Hospital, 50134 Florence, Italy; stefano.mombelli@unifi.it; 2 Neurology Unit, Santo Stefano Hospital, 59100 Prato, Italy; 3 Neurology Unit, Department of Clinical and Experimental Sciences, University of Brescia, 25123 Brescia, Italy; 4 IRCCS Istituto delle Scienze Neurologiche di Bologna, UOC Neurologia e Rete Stroke Metropolitana, Ospedale Maggiore, 40100 Bologna, Italy; 5 Neurology Unit, Stroke Unit, Department of Neuroscience, Santa Maria delle Croci Hospital, AUSL Romagna, 48121 Ravenna, Italy; 6 Neurology Unit, Stroke Unit, Azienda Unità Sanitaria Locale-IRCCS di Reggio Emilia, 42123 Reggio Emilia, Italy; 7 Neuroradiology Unit, Ospedale Santa Maria della Misericordia, AULSS5 Polesana, 45100 Rovigo, Italy; 8 Neurology Unit, Fondazione Policlinico Universitario Agostino Gemelli IRCCS, 00168 Rome, Italy; 9 U.C. Malattie Cerebrovascolari e Stroke Unit, IRCCS Fondazione Mondino, 27100 Pavia, Italy; 10 Stroke Unit, Careggi University Hospital, 50134 Florence, Italy

**Keywords:** stroke, small vessel disease, small subcortical infarct, hyperglycemia

## Abstract

**Introduction:** Hyperglycemia is a common finding in patients with acute ischemic stroke and has been associated with poor outcomes, but its relationship with stroke severity at presentation is unclear. We investigated the associations between blood glucose levels and stroke severity in patients with acute small subcortical infarct (SSI). **Methods:** We retrospectively reviewed records of consecutive patients with acute SSI admitted to six Stroke Units. Stroke severity was assessed using the National Institutes of Health Stroke Scale (NIHSS). Outcomes of interest were as follows: NIHSS ≥ 7 at admission; increasing NIHSS quartiles at discharge. Independent associations between baseline glucose levels and stroke severity at presentation and discharge were assessed with logistic and ordinal regression, adjusting for age, sex, hypertension, diabetes, smoke exposure, and functional independence. **Results:** We included 792 patients: mean (±SD) age of 69.8 (±13) years, 548 (69%) males, median (IQR) NIHSS = 4(2–6). The mean blood glucose level at admission was 130 (±59) mg/dL. Patients with moderate–severe NIHSS had higher baseline blood glucose levels compared to those with lower NIHSS (148 mg/dL vs. 125 mg/dL, *p* < 0.001). After adjusting for confounders, glucose levels ≥ 130 mg/dL and the highest blood glucose quartile were associated with baseline NIHSS ≥ 7 (OR = 1.61; 95%CI = 1.04–2.51; OR = 1.80; 95%CI = 1.13–2.89, respectively). Glucose levels ≥ 130 mg/dL and the highest blood glucose quartile were associated with a shift towards higher NIHSS at discharge (cOR = 1.54; 95%CI = 1.11–2.14; cOR = 1.61; 95%CI = 1.14–2.29, respectively). **Conclusions:** In patients with acute SSI, higher blood glucose levels are associated with greater stroke severity at both admission and discharge, suggesting a potential biological link between blood glucose and the severity of SSI.

## 1. Introduction

Small subcortical infarct (SSI) is a subtype of ischemic stroke caused by small vessel disease affecting small penetrating arteries supplying deep brain structures. SSI is often associated with cardiovascular risk factors such as hypertension and diabetes, which may contribute to vascular damage (lipohyalinosis and microatheroma formation) [1,2,3]. Despite the relatively favourable short-term prognosis, with a low early mortality and marginal functional disability at hospital discharge, SSI is associated with an elevated risk of mortality, stroke recurrence, and cognitive decline [4,5]. Stroke severity, even at presentation, is a strong predictor of poor prognosis. Identifying factors associated with greater initial severity may allow early targeted interventions aimed at mitigating their adverse impact on clinical outcomes [6].

Hyperglycemia is frequently observed in patients with acute ischemic stroke, affecting approximately 40% of patients at admission, and occurs among all ischemic stroke subtypes [7,8]. Hyperglycemia triggers a cascade of biochemical pathways in endothelial cells [9], leading to pathological changes such as those observed in diabetic complications [10,11]. While most evidence derives from models of chronic hyperglycemia, several of these pathways are also activated acutely, contributing to early cerebrovascular dysfunction in ischemic stroke [12]. In patients with acute ischemic stroke treated with Intravenous thrombolysis, admission hyperglycemia has been associated with larger ischemia, reduced functional and cognitive outcomes, and increased risk of mortality [13]. In large and cortical infarcts, admission hyperglycemia has been consistently associated with worse outcomes; however, the effect of high blood glucose levels in SSI remains unclear [14]. While some reports suggested a potential detrimental effect, particularly on SSI patients [15], other studies have proposed a possible protective role on long-term follow-up [16,17]. Notwithstanding these findings, evidence regarding a putative effect of hyperglycemia on admission stroke severity is lacking. This relationship could be relevant in understanding the pathophysiology and mechanisms of stroke severity in acute SSI, given the association between stroke severity and outcomes in this stroke subtype. Therefore, in patients with acute SSI, we aimed to investigate the association between blood glucose level (BGL) at admission and stroke severity both at admission and at discharge.

## 2. Materials and Methods

We retrospectively reviewed the records of consecutive patients with acute SSI admitted at six Italian Stroke Units (Careggi University Hospital, Florence; Azienda Unità Sanitaria Locale, Reggio Emilia; ASST Spedali Civili di Brescia, Ospedale Maggiore, Bologna; Fondazione Policlinico Universitario A. Gemelli IRCCS, Rome; and IRCCS Fondazione Mondino, Pavia) in the period between 2009 and 2023. Patients were eligible for inclusion if they (1) presented with a classical lacunar stroke syndrome, as defined by the Oxfordshire Community Stroke Project (OCSP) [18]; (2) showed a small subcortical lesion in the territory of a perforating artery, located within one of the following supratentorial or infratentorial territories (lenticulostriate branches, basilar perforators, or posterior cerebral/ posterior communicating artery territories); (3) had an ischemic lesion detectable on either computed tomography (CT) or magnetic resonance imaging (MRI); and (4) were admitted to the hospital within 72 h from symptom onset. We excluded individuals with (1) cortical or multiple ischemic lesions, (2) a clearly identified non-small-vessel etiology, or (3) a delay exceeding 72 h between symptom onset and hospital presentation. The institutional review boards and ethical committees of each participating centre approved the study (N. 13068/OSS, approved in August 2018 and subsequently amended throughout the years, with the last amendment in January 2024). Informed consent was obtained within 48 h of hospital admittance, or it was waived by the institutional review board according to each centre’s practice. This research was conducted ethically in accordance with the World Medical Association Declaration of Helsinki. Baseline demographic and clinical characteristics were systematically recorded for all enrolled patients, including age, sex, and vascular risk factors. Hypertension was defined as either the current use of antihypertensive medication or elevated blood pressure on admission (SBP > 140 mmHg or DBP > 90 mmHg). Diabetes mellitus was defined by ongoing glucose-lowering treatment or by admission blood glucose levels meeting diagnostic thresholds (fasting glucose ≥ 126 mg/dL or random glucose ≥ 200 mg/dL). Hyperlipidemia was defined as the use of lipid-lowering therapy or a fasting total cholesterol concentration > 200 mg/dL during hospitalization. Information on alcohol consumption; current smoking status; prior cardiovascular or cerebrovascular disease (ischemic heart disease, peripheral artery disease, atrial fibrillation, previous stroke or transient ischemic attack); pre-stroke medications (antiplatelets, anticoagulants, antihypertensives, lipid-lowering agents); and premorbid functional status assessed with the modified Rankin Scale (mRS) was also collected. At hospital admission, systolic and diastolic blood pressure values and blood glucose level (BGL) were measured. Stroke severity was assessed by a stroke neurologist using the National Institutes of Health Stroke Scale (NIHSS). Lacunar syndromes were categorized according to the Oxfordshire Community Stroke Project (OCSP) classification [18], including pure motor hemiparesis, pure sensory stroke, sensorimotor stroke, and ataxic hemiparesis. All patients underwent at least a 12-lead electrocardiogram and transthoracic echocardiography to identify signs of hypertensive heart disease (left-ventricular hypertrophy or impaired diastolic relaxation) and to exclude relevant structural cardiac abnormalities. Neuroimaging included a brain CT scan at admission, followed by a repeat CT after 24–36 h. When the ischemic lesion was not visible on CT or based on the clinical judgement of the treating neurologist, MRI (1.5 or 3 T) was performed. The MRI protocol included, at minimum, diffusion-weighted imaging (DWI), FLAIR, and T1-weighted, T2-weighted, and T2* sequences. All imaging studies were retrospectively reviewed by stroke neurologists or a neuroradiologist, who were blinded to clinical data. When the infarct was visible on CT or MRI, the SSI dimension was measured on an axial plane. Outcome measures were (1) the stroke severity at admission, defined as NIHSS ≥ 7, used as a dichotomous indicator of moderate/severe stroke (the NIHSS cut-off of <7 for identifying SSI was proposed according with previous studies [19,20]); (2) neurological status at discharge, assessed through NIHSS score quartiles, allowing stratification of patients according to the degree of residual neurological impairment and Delta NIHSS, defined as the difference between NIHSS at admission and at discharge; (3) stroke progression, defined as an increase of at least 2 points in NIHSS score in the first 72 h since hospital admission; (4) length of hospital stay; and (5) discharge destination (home, rehabilitation facility).

## 3. Statistical Analysis

Descriptive statistics were used to summarize the general characteristics of the study population. Categorical variables were reported as absolute frequencies and percentages (N, %). For continuous variables, distribution normality was assessed using the Kolmogorov–Smirnov test, and data were presented accordingly. Comparisons between groups were performed using Pearson’s χ^2^ test for categorical data and the Mann–Whitney U test for continuous variables. To explore the associations between blood glucose levels and stroke severity, we dichotomized glucose levels using (1) the mean of the distribution as a cut-off and (2) the quartiles of the distribution. We assessed independent associations between baseline blood glucose levels and stroke severity at presentation and discharge with logistic and ordinal regression, respectively, adjusting for age, sex, hypertension, diabetes mellitus, smoke exposure, and pre-stroke functional independence (mRS 0–2). We expressed the association with odds ratio (OR) and 95% confidence interval (95%CI). Differences were considered statistically significant at a level of *p* < 0.05 for a two-sided test for independent associations. Statistical analysis was performed with SPSS for Mac software (version 26.0; SPSS, ArmonkIBM Corp, Armonk, NY, USA).

## 4. Results

During the study period, 876 patients received a diagnosis of acute clinical SSI. Among them, 84 (9%) showed no detectable ischemic lesion on either CT or MRI and were therefore excluded. The final cohort comprised 792 patients, of whom 639 (81%) presented with an NIHSS score < 7, while 153 (19%) had an NIHSS score ≥ 7. Mean (±SD) age was 69.8 (±12.8) years, 548 (69%) were males, 201 (25%) received rt-PA, 541 (68%) had hypertension, and 186 (24%) had diabetes; 564 (89%) patients underwent MR, while in 302 (38%), the infarct lesion was visible on CT. Median (IQR) NIHSS at admission was 4 (2–6), while at discharge, it was 2 (1–4), and the mean blood glucose level at admission was 129.5 (±59.0) mg/dL. Patients with NIHSS ≥ 7 more frequently had newly diagnosed hypertension (24% vs. 17%, *p* = 0.041), more frequently received rt-PA (46% vs. 21%, *p* < 0.001), had a higher blood glucose level at admission (147.99 mg/dL vs. 125.08 mg/dL, *p* < 0.001), and had a larger axial diameter (12.14 mm vs. 9.98 mm, *p* < 0.001) (Table 1). Stroke progression occurred in 13% of patients and the median NIHSS score at discharge was 2 (1–4). Patients with moderate/severe NIHSS more frequently experienced progression (18% vs. 11%, *p* = 0.038), a longer hospital stay (6.8 days vs. 6.1 days, *p* = 0.013), and more frequently required post-discharge rehabilitation (67% vs. 33%, *p* < 0.001) (Table 2).

We dichotomized BGL using the cutoff of 130 mg/dL (mean value of the whole study population). Patients with moderate–severe NIHSS more frequently had BGL ≥ 130 mg/dL (40% vs. 27%, *p* = 0.002) compared with those with lower NIHSS. After adjustment for confounders, higher BGL was independently associated with increased odds of severe stroke at admission (aOR = 1.61; 95%CI = 1.04–2.51). When stratified by BGL quartiles, patients in the highest quartile (>137 mg/dL) more frequently presented with NIHSS ≥ 7 (22 vs. 35%, *p* = 0.003); this association was confirmed in multivariate analysis (aOR = 1.80; 95%CI = 1.13–2.89) (Table 3). Similarly, patients with BGL ≥ 130 mg/dL and those within the highest BGL quartile showed a shift towards worse NIHSS at discharge (cOR = 1.54, 95%CI = 1.11–2.14; cOR = 1.61, 95% CI 1.14–2.29, respectively) in adjusted ordinal analysis (Table 4). BGL ≥ 130 mg/dL was associated with length of stay ≥ 6 days (aOR = 1.47, 95% CI 1.02–2.11), while BGL ≥ 130 mg/dL and in the highest quartile were both associated with discharge with rehabilitation facilities (aOR = 1.90, 95% CI 1.31–2.75, 1.78 95% CI 1.20–2.65, respectively), but not with stroke progression (Table 4).

## 5. Discussion

In this study, we investigated the association between blood glucose level and stroke severity in patients with small subcortical infarct. We further investigated whether blood glucose was related to stroke progression, length of hospital stay, and destination at discharge. We found that a higher blood glucose level at admission was independently associated with greater stroke severity at both presentation and discharge, suggesting a potential role of hyperglycemia in stroke severity in the context of stroke caused by small vessel disease. Moreover, a higher blood glucose level was associated with a longer hospital stay and discharge to rehabilitation facilities.

The association between admission hyperglycemia and poor outcomes in ischemic stroke has been widely investigated in non-small subcortical infarct. Hyperglycemia in acute ischemic stroke treated with IV-tPA has been related to higher mortality, symptomatic hemorrhage occurrence, and poor 90-day functional outcomes [16,21,22,23]. Nonetheless, data specifically addressing SSI lack consistency. Previous studies on SSI suggested no adverse relationship between hyperglycemia and outcomes or a paradoxically favourable effect. Analysis from large multicenter datasets showed an association between elevated glucose and worse prognosis in non-small subcortical infarct, whereas there was no relation in small subcortical infarct [15]. In some cohorts, moderate hyperglycemia was associated with good functional outcomes in patients with acute SSI, possibly reflecting preserved collateral perfusion or distinct metabolic demands of small deep infarcts [17]. More recently, it has been demonstrated that the prognostic value of early glycemic profile is dependent on the stroke subtype, with negative associations in non-SSI but neutral or absent effects in SSI [24]. However, such studies often suffered from small sample sizes, limited glucose profiling, or lack of adjustment for meaningful confounders and the association with admission stroke severity has been scarcely investigated. Our findings diverge from those studies, revealing that higher glucose at presentation, namely ≥130 mg/dL, was consistently associated with higher stroke severity at admission and discharge, lending support to the hypothesis that acute high blood glucose level independently contributes to neurological severity in SSI. Given that both NIHSS admission and blood glucose levels were taken before rt-PA administration, we could infer that this relation is independent of rt-PA itself. We did not find any association with stroke progression, which has been previously associated with length of stay and destination at discharge [25]. Rather, we found that higher BGL increased the length of hospital stay and need for rehabilitation at discharge, reflecting the main finding of our study, i.e., the association between high BGL and stroke severity. The biological mechanisms underlying our results may comprise a wide range of mechanisms. Endothelial dysfunction, mediated by reduced nitric oxide availability and a proinflammatory–prothrombotic shift, has been linked to elevated glucose levels. Higher glucose levels may enhance oxidative stress through mitochondrial superoxide activation, and impaired glucose transporter regulation in pericytes may further disrupt microvascular homeostasis, eventually leading to impaired perfusion and neurovascular coupling [8,9,10,11,12,26,27]. These pathways are detrimental in the context of SSI, where ischemic injury is linked to endothelial dysfunction [28,29]. Furthermore, in patients with small vessel disease, there is an impairment of cerebral perfusion in white matter in the acute phase of stroke [30], and higher BGL may enhance ischemic damage by further compromising microvascular integrity and endothelial dysfunction, increasing blood–brain barrier permeability and triggering local proinflammatory responses [31,32]. As a consequence, both metabolic and vascular stress in subcortical areas may partially explain the observed association between higher admission glucose levels and increased stroke severity.

Our findings suggest that admission fasting blood glucose levels may represent a simple and clinically valuable marker of stroke severity in patients with acute SSI and perhaps a potential and feasible therapeutic target. Although large randomized controlled trials of glucose control did not demonstrate an improvement in outcome and were associated with an increased risk of hypoglycemia [23,33], it should be noted that they were run in unselected ischemic stroke populations, whereas our findings highlight the potential value of tailored glucose-guided interventions in the selected population of patients with SSI. Future prospective studies may clarify whether hyperacute glucose control management may improve clinical outcomes in this stroke subtype. Incorporating serial glucose measurements alongside HbA1c would help differentiate between acute and chronic glycemic disturbances, clarifying their prognostic implications. Perfusion imaging showed promising results in the identification of small subcortical lesions [34,35], and the effect of glucose levels on perfusion maps on this stroke subtype might provide further insights into pathophysiology and prognosis. Finally, although we did not find any association with short-term outcomes, an extended follow-up is needed to investigate the long-term consequences of higher BGL on stroke recurrence, cognitive outcomes, and functional recovery in this population.

This study has limitations. Firstly, the retrospective design, based on the review of medical records, may introduce assessment bias due to variability in data and documentation. For example, we did not have information regarding the time between the onset of symptoms and the assessment of BGL; rather, we included patients from a period within 72 h of symptoms onset to intercept patients who already had early neurological deterioration. Secondly, selection bias cannot be excluded, as data were derived exclusively from patients admitted to Stroke Units; individuals managed in other hospital wards or intensive care settings were not included, potentially limiting the generalizability of our findings. Finally, only a single baseline glucose measurement was available, which prevented the evaluation of glycemic trends over time, including potential fluctuations that might carry additional prognostic value. The strengths of the study are its multicenter design and the rigorous application of both clinical and imaging criteria to ensure a homogeneous population of patients with small subcortical infarct. The analysis of blood glucose levels both as a dichotomous variable and as quartiles allowed for a comprehensive evaluation of their relationship with stroke severity and revealed a dose–response effect, supporting the causal relation between glucose levels and stroke severity.

## 6. Conclusions

In patients with acute small subcortical infarct, higher admission blood glucose is independently associated with more severe neurological deficits at presentation and discharge, supporting the suggestion of a biological relationship between higher blood glucose level and stroke severity. Further prospective studies are needed to evaluate the long-term consequences of blood glucose levels in such a population and whether the management of blood glycemia can modify outcomes in this subset of stroke patients.

## Figures and Tables

**Table 1:** Baseline characteristics of patients with NIHSS ≥ 7 or <7.

	Total n = 792	NIHSS < 7 n = 639	NIHSS ≥ 7 n = 153	*p*
Age, years, mean (±SD)	69.79 (±12.8)	69.69 (±13.1)	70.18 (±12.1)	0.673
Sex, male	548 (69)	450 (70)	98 (64)	0.125
Previous TIA/stroke	151 (19)	118 (18)	33 (22)	0.975
Previous mRS (0–2)	730 (92)	597 (93)	133 (87)	0.007
Hypertension	541 (68)	442 (69)	99 (65)	0.286
Diabetes Mellitus	186 (24)	141 (22)	45 (30)	0.054
Hyperlipidemia	257 (32)	208 (33)	49 (32)	0.901
Atrial Fibrillation	37 (5)	27 (4)	10 (7)	0.224
Smoking	294 (37)	239 (38)	55 (36)	0.728
Alcohol	84 (11)	62 (10)	22 (14)	0.093
NIHSS at admission, median, (IQR)	4 (2–6)	3 (2–4)	8 (7–11)	<0.001
Systolic BP, mmHg, mean (±SD)	162.67 (±26.1)	162.47 (±25.9)	163.49 (±27.3)	0.667
Diastolic BP, mmHg, mean (±SD)	88.33 (±15.6)	88.31 (±15.4)	88.41 (±16.8)	0.946
Blood Glucose Level at admission, mg/dL, mean (±SD)	129.51 (±59.0)	125.08 (±51.8)	147.99 (±80.2)	<0.001
Blood Glucose Level ≥ 130 mg/dL	236 (30%)	175 (24%)	61 (40%)	0.002
Blood Glucose Level quartiles				0.003
1st quartile (<96 mg/dL)	207 (26%)	167 (26%)	40 (26%)	
2nd quartile (96–110 mg/dL)	192 (24%)	167 (26%)	25 (16%)	
3rd quartile (110–137 mg/dL)	196 (25%)	162 (25%)	34 (22%)	
4th quartile (>137 mg/dL)	197 (25%)	143 (22%)	54 (35%)	
Creatinine at admission, mg/dL, mean (±SD)	0.92 (±0.5)	0.93 (±0.5)	0.86 (±0.3)	0.096
rt-PA	201 (25)	131 (21)	70 (46)	<0.001
CT	302 (38)	224 (35)	78 (51)	<0.001
MR	564 (89)	468 (91)	96 (84)	0.023
Axial diameter, mm, mean (±SD)	10.41 (±5.4)	9.98 (±5.3)	12.14 (±5.4)	<0.001

All values are to be considered as N (%) unless otherwise specified. SD = standard deviation; IQR = interquartile range; BP = blood pressure; TIA = transient ischemic attack; mRS = modified Rankin Scale; NIHSS = National Institute of Health Stroke Scale; rt-PA= tissue plasminogen activator; CT = computed tomography; MR = magnetic resonance imaging. Underlining is used to highlight variables with a *p*-value < 0.05.

**Table 2:** In-hospital clinical course and at-discharge parameters in patients with NIHSS ≥ 7 or <7.

	Total n = 792	NIHSS < 7 n = 639	NIHSS ≥ 7 n = 153	*p*
Progression	100 (13)	73 (11)	27 (18)	0.037
Length of Hospital stay, days, mean (±SD)	6.21 (±3.7)	6.07 (±3.2)	6.79 (±4.0)	0.013
Neurological status at discharge, median, (IQR)	2 (1–4)	2 (1–3)	5 (3–8)	<0.001
Delta NIHSS, median, (IQR)	1 (0–3)	1 (0–2)	2 (4–6)	<0.001
Destination				<0.001
Home	477 (61)	427 (67)	50 (33)	
Rehab Facilities	313 (40)	211 (33)	102 (67)	

All values are to be considered as N (%) unless otherwise specified. SD = standard deviation IQR = interquartile range; NIHSS = National Institute of Health Stroke Scale. Underlining is used to highlight variables with a *p*-value < 0.05.

**Table 3:** Association between blood glucose level (BGL) and stroke severity (NIHSS ≥ 7) at admission.

Variables	Crude OR (95% CI)	Adjusted OR (95% CI)
BGL (mg/dL)	1.01 (1.00–1.01)	1.01 (1.00–1.01)
BGL ≥ 130 mg/dL	1.76 (1.22–2.54)	1.61 (1.04–2.51)
BGL 4th quartile (>137 mg/dL)	1.89 (1.29–2.77)	1.80 (1.13–2.89)

Independent associations between BGL and stroke severity at admission were analysed using logistic regression, adjusting for age, sex, hypertension, diabetes mellitus, smoke exposure, and pre-stroke functional independence. BGL = blood glucose level; OR = odds ratio; CI = confidence interval.

**Table 4:** Association between blood glucose level (BGL) and outcomes at discharge.

Variables	Outcome	Crude OR (95% CI)	Adjusted OR (95% CI)
Neurological status at discharge (NIHSS quartiles)			
BGL (mg/dL)		1.00 (0.99–1.00)	-
BGL ≥ 130 mg/dL		1.75 (1.33–2.30)	1.54 (1.11–2.14)
BGL 4th quartile (>137 mg/dL)		1.83 (1.37–2.46)	1.61 (1.14–2.29)
Progression			
BGL (mg/dL)		1.00 (0.99–1.00)	-
BGL ≥ 130 mg/dL		1.32 (0.85–2.05)	-
BGL 4th quartile (>137 mg/dL)		1.01 (0.66–1.73)	-
Length of stay ≥ 6 days			
BGL (mg/dL)		1.00 (0.99–1.00)	-
BGL ≥ 130 mg/dL		1.60 (1.18–2.18)	1.47 (1.02–2.11)
BGL 4th quartile (>137 mg/dL)		1.48 (1.07–2.05)	1.32 (0.89–1.95)
Rehabilitation at discharge			
BGL (mg/dL)		1.00 (0.99–1.00)	-
BGL ≥ 130 mg/dL		2.11 (1.55–2.88)	1.90 (1.31–2.75)
BGL 4th quartile (>137 mg/dL)		2.03 (1.46–2.81)	1.78 (1.20–2.65)

Independent associations between BGL and neurological status at discharge were analyzed using ordinal regression, adjusting for age, sex, hypertension, diabetes mellitus, smoke exposure, and pre-stroke functional independence, while associations between BGL and length of stay ≥6 days and rehabilitation at discharge were analyzed using logistic regression, adjusting for age, sex, hypertension, diabetes mellitus, smoke exposure, and pre-stroke functional independence. BGL = blood glucose level; NIHSS = National Institute of Health Stroke Scale; OR = odds ratio; CI = confidence interval.

## Data Availability

The original contributions presented in this study are included in the article. Further inquiries can be directed to the corresponding author.
